# Predicting 7-day unplanned readmission in elderly patients with coronary heart disease using machine learning

**DOI:** 10.3389/fcvm.2023.1190038

**Published:** 2023-08-08

**Authors:** Xuewu Song, Yitong Tong, Yi Luo, Huan Chang, Guangjie Gao, Ziyi Dong, Xingwei Wu, Rongsheng Tong

**Affiliations:** ^1^ Department of Pharmacy, Sichuan Provincial People’s Hospital, University of Electronic Science and Technology of China, Chengdu, China; ^2^ Chinese Academy of Sciences Sichuan Translational Medicine Research Hospital, Chengdu, China; ^3^ Chengdu Second People’s Hospital, Chengdu, China

**Keywords:** readmission, machine learning, coronary heart disease, elderly, predict

## Abstract

**Background:**

Short-term unplanned readmission is always neglected, especially for elderly patients with coronary heart disease (CHD). However, tools to predict unplanned readmission are lacking. This study aimed to establish the most effective predictive model for the unplanned 7-day readmission in elderly CHD patients using machine learning (ML) algorithms.

**Methods:**

The detailed clinical data of elderly CHD patients were collected retrospectively. Five ML algorithms, including extreme gradient boosting (XGB), random forest, multilayer perceptron, categorical boosting, and logistic regression, were used to establish predictive models. We used the area under the receiver operating characteristic curve (AUC), accuracy, precision, recall, the F1 value, the Brier score, the area under the precision-recall curve (AUPRC), and the calibration curve to evaluate the performance of ML models. The SHapley Additive exPlanations (SHAP) value was used to interpret the best model.

**Results:**

The final study included 834 elderly CHD patients, whose average age was 73.5 ± 8.4 years, among whom 426 (51.08%) were men and 139 had 7-day unplanned readmissions. The XGB model had the best performance, exhibiting the highest AUC (0.9729), accuracy (0.9173), F1 value (0.9134), and AUPRC (0.9766). The Brier score of the XGB model was 0.08. The calibration curve of the XGB model showed good performance. The SHAP method showed that fracture, hypertension, length of stay, aspirin, and D-dimer were the most important indicators for the risk of 7-day unplanned readmissions. The top 10 variables were used to build a compact XGB, which also showed good predictive performance.

**Conclusions:**

In this study, five ML algorithms were used to predict 7-day unplanned readmissions in elderly patients with CHD. The XGB model had the best predictive performance and potential clinical application perspective.

## 1. Introduction

Coronary heart disease (CHD) is a common noncommunicable chronic cardiovascular disease in the elderly (1). Many elderly CHD patients require repeated hospitalizations due to poor disease control. Readmission refers to the patient returning to the hospital for the same or related treatment within a certain period after discharge (2). Readmission rates have become an important hospital performance measure for healthcare efficiency and quality improvement because unplanned readmission often means the failure of the initial intervention, especially for short-term readmission patients (3). Because of impaired mobility in elderly patients, repeated readmissions may be a difficult experience for patients and their families (4). In addition, frequent readmissions can increase the financial burden of patients, reduce their quality of life, and cause excessive consumption of medical resources. Therefore, it is of great significance to reduce the readmission of elderly CHD patients.

To that end, screening tools already exist to identify patients who may be readmitted to the hospital (4–7). However, these screening tools were developed primarily from data of 30-day or 1-year readmitted patients, which limited the accuracy of these tools in assessing patients’ 7-day unplanned readmission. Studies have shown that 84% of 7-day readmissions are avoidable (8). Therefore, developing a predictive model to assess the risk of readmission is the key to reducing 7-day unplanned readmission. Further, predicting the high risk of 7-day readmission may help avoid short-term unplanned readmission by targeted interventions. However, to our knowledge, there have been no established protocols for 7-day unplanned readmission in elderly patients with CHD.

A careful evaluation of the rehospitalization risk of elderly CHD patients plays a fundamental role in the clinical management of each patient. In recent years, the application of machine learning (ML) algorithms to predict clinical events has been actively conducted (9–11), and the development of a complicated and reliable classification tool has become possible. Therefore, we hypothesized that combining ML algorithms with patients’ basic information might make it possible to produce reliable prediction models to predict the 7-day unplanned readmission of elderly CHD patients. The purpose of this study was to collect patients’ basic information from the electronic medical record (EMR) system to establish an ML model for the prediction of unplanned readmission within 7 days of discharge in elderly patients with CHD.

## 2. Materials and methods

### 2.1. Study population and data source

We retrospectively collected the data of elderly CHD patients who underwent 7-day readmission in Sichuan Provincial People’s Hospital from July 2018 to June 2020. Also, we matched the non-readmission patients to the 7-day readmission patients by the ratio of 5:1. CHD, as a principal diagnosis, was confirmed by using the International Classification of Disease (ICD-10) codes (I20–I25). The patients aged <60 years, transferred to other hospitals, or readmitted by some specific treatments such as hemodialysis or radiation therapy will be excluded. We also excluded the patients with missing severe data or who died in the hospital. We collected the general information, records of diagnoses, medications, and comorbidities of the patients (7). For multiple laboratory results, we selected the last results of patients before discharge. This study was approved by the Ethics Committee of the Sichuan Academy of Medical Sciences and Sichuan Provincial People’s Hospital. Due to the retrospective nature of the study, informed consent was waived. Also, we hid the patients’ personal information during the study.

### 2.2. Data preprocessing

First, all variables were blinded to eliminate subjective influence. Then, categorical variables were represented by 0 and 1, continuous variables were standardized using the Z-score, and laboratory examinations were represented by 1, 2, and 3 (1, below the normal range; 2, within the normal range; and 3, above the normal range). The variables with missing data >90%, a single value occupying >90%, or the coefficient of variation <0.1 were deleted. Finally, we used random forest (RF) to replace the missing value, and Lasso was used for variable selection.

### 2.3. Machine learning algorithms

To establish the best prediction model, we used five representative ML algorithms, including extreme gradient boosting (XGB), RF, multilayer perceptron (MLP), categorical boosting (CB), and logistic regression (LR), as prediction model-based algorithms.

XGB is an ensemble classifier based on regression tree, which has the characteristics of short training time and high precision (12). XGB uses residuals to improve the model and adds an internal regularization to prevent overfitting and enhance the robustness of the model.

RF is also an ensemble classifier composed of hundreds to thousands of decision trees (13). The final classification result is determined by the mode of the output result by each tree. Moreover, this classifier has strong stability and robustness for small amounts of noise and outliers.

MLP is an ML algorithm developed by feedforward neural networks, which are composed of ordered layers comparable to human neuron processing (14). Structurally, the MLP model comprises an input layer, an output layer, and one or more hidden layers (15).

CB is an ML classification technique based on oblivious trees, which can deal with classification problems efficiently and reasonably (16). CB solves the problem of gradient bias and prediction shift to reduce the overfitting risk and improve the accuracy and generalization ability of the algorithm.

An LR model describes and estimates the relationship between one or more independent variables and one binary dependent variable (17). LR is used to compute the probability of an occurrence of binary outcomes (18). It has a powerful interpretation and has been widely used in diverse areas of medical research.

### 2.4. Model establishment

XGB, MLP, RF, LR, and CB were used to build the prediction models. The model establishment was as follows. All patients were randomly divided into a training set and a test set in the proportion of 8:2. The training set was used to build the classification models, and the test set was used to evaluate the predictive performance. Moreover, the borderline synthetic minority oversampling technique (SMOTE) (19) was used to solve the issues associated with the imbalanced data in the training set. Borderline SMOTE is an improved oversampling algorithm over SMOTE. Also, borderline SMOTE can generate new data from borderline data, thereby improving the category distribution of samples. Meanwhile, we trained the ML models on original data and evaluated their predictive performance on the test set.

### 2.5. Model evaluation

To evaluate the predictive performance of the ML models, we calculated five representative performance evaluation measures, including area under the receiver operating characteristic curve (AUC), accuracy, precision, recall, and F1 value. Meanwhile, as precision and recall are more meaningful in clinical practice, we also computed the area under the precision-recall curve (AUPRC) to assess the model performance. The model’s calibration was evaluated by the Brier score and calibration plot. The model was considered to have favorable calibration when the Brier score was ≤0.25 (20). SHapley Additive exPlanations (SHAP) values were used to measure the contribution of each variable to the best model.

### 2.6. Sample size assessment

We chose a repeated bootstrapping method to evaluate the appropriateness of sample size. First, we randomly selected 10%, 20%, and 30%–100% subsets from the training set by 100 times, respectively. Then, these subsets were used to establish prediction models combined with the best ML algorithm selected by model evaluation. Finally, we calculated the AUC of the test set based on the established models to assess the sample size.

### 2.7. Statistical analysis

Chi-square tests were used to analyze categorical variables expressed as counts and percentages. Continuous variables were expressed as means ± standard deviations or medians with first to third quartiles (median, Q1–Q3). We used the *t*-test or Mann–Whitney test to analyze the statistical significance. *P*-values <0.05 were considered statistically significant. Statistical analyses were performed by using SPSS software version 25 (IBM SPSS Statistics, IBM Corporation, Armonk, NY, United States). Model building was implemented using the stats and sklearn packages in Python (Version 3.8).

## 3. Results

### 3.1. Study population

**Figure 1:**
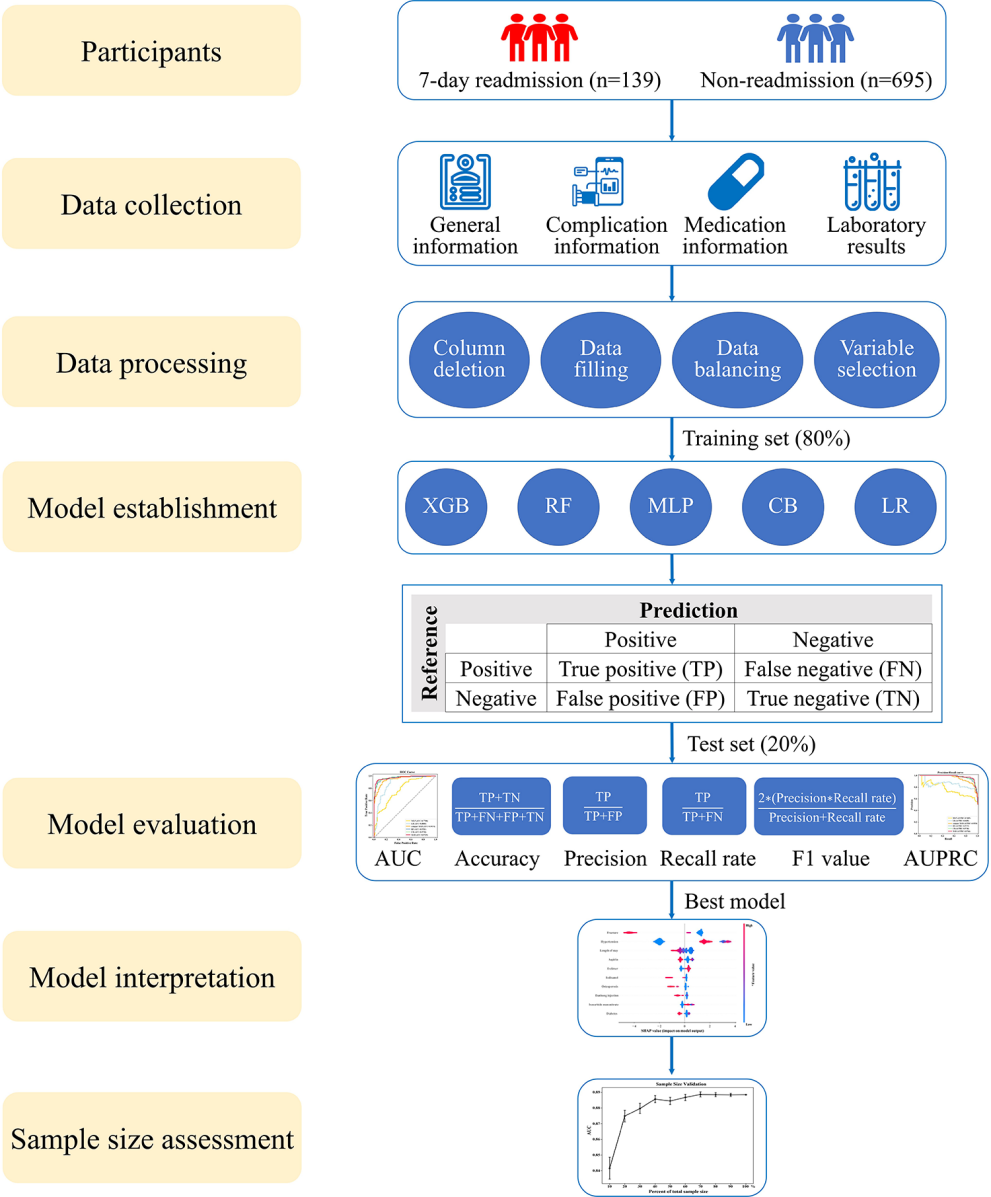
Research roadmap of the study. XGB, extreme gradient boosting; RF, random forest; MLP, multilayer perceptron; CB, categorical boosting; LR, logistic regression; AUC, area under the receiver operating characteristic curve; AUPRC, area under the precision-recall curve.

We enrolled 139 patients who underwent 7-day unplanned readmission and matched the non-readmission patients (695) by a ratio of 5:1 in this study. A total of 834 elderly CHD patients were included, of which 426 (51.1%) were men and 408 (48.9%) were women. The average age was 73.5 ± 8.4 years, and the length of stay was 6 (3, 11) days. The general information of the patients is presented in Table 1. The whole process of the study is shown in Figure 1.

**Table 1:** Baseline characteristics of 7-day readmission vs. non-readmission patients.

Feature	7-day readmission (*n* = 139)	Non-readmission (*n* = 695)	*P*-values
Age, years	76 (68, 82)	72 (66, 78)	<0.01
Gender			0.853
Male	72 (51.8%)	354 (50.9%)	
Female	67 (48.2%)	341 (49.1%)	
Length of stay	7 (3, 12)	6 (3, 10)	0.080
Hypertension			<0.001
Yes	91 (65.5%)	114 (16.4%)	
No	48 (34.5%)	581 (83.6%)	
Diabetes			0.701
Yes	50 (36.0%)	262 (37.7%)	
No	89 (64.0%)	433 (62.3%)	
Fracture			<0.001
Yes	10 (7.2%)	403 (58.0%)	
No	129 (92.8%)	292 (42.0%)	
Heart failure			0.468
Yes	32 (23.0%)	141 (20.3%)	
No	107 (77.0%)	554 (79.7%)	
Osteoporosis			<0.001
Yes	20 (14.4%)	223 (32.1%)	
No	119 (85.6%)	472 (67.9%)	
Aspirin			<0.001
Yes	53 (38.1%)	379 (54.5%)	
No	86 (61.9%)	316 (45.5%)	
Danhong injection			<0.01
Yes	30 (21.6%)	240 (34.5%)	
No	109 (78.4%)	455 (65.5%)	
Isosorbide mononitrate			0.248
Yes	36 (25.9%)	149 (21.4%)	
No	103 (74.1%)	546 (78.6%)	
Iodixanol			<0.001
Yes	7 (5.0%)	186 (26.8%)	
No	132 (95.0%)	509 (73.2%)	
Laboratory results			
GGT, U·L^−1^	27 (16, 44)	23 (17, 36)	0.126
ALB, g·L^−1^	38.0 (34.6, 42.0)	40.2 (37.1, 43.4)	<0.001
ALT, U·L^−1^	18 (13, 27)	22 (15, 32)	<0.05
TG, mmol·L^−1^	1.22 (0.94, 1.79)	1.38 (0.94, 1.91)	0.109
eGFR, ml·min^−1^	78.8 (56.3, 89.1)	84.8 (67.0, 93.4)	<0.01
Urea, mmol·L^−1^	6.42 (5.01, 9.13)	5.98 (4.94, 7.79)	0.201
AST, U·L^−1^	27 (22, 34)	27 (22, 35)	0.577
TC, mmol·L^−1^	3.74 (2.92, 4.32)	3.98 (3.23, 4.77)	<0.05
TBIL, µmol·L^−1^	13.5 (10.3, 17.7)	13.6 (10.2, 18.1)	0.733
TP, g·L^−1^	67.2 (61.5, 72.5)	68.6 (63.9, 72.9)	0.090
WBC, *10^9^·L^−1^	6.4 (5.1, 8.1)	6.2 (5.2, 7.7)	0.370
RBC, *10^12^·L^−1^	4.1 (3.6, 4.6)	4.3 (3.8, 4.6)	<0.05
HCT, %	38.6 (33.5, 41.5)	39.4 (35.7, 42.6)	<0.05
HGB, g·L^−1^	123 (109, 137)	130 (117, 141)	<0.01
Cholinesterase, KU·L^−1^	6.5 (5.1, 8.0)	7.4 (6.1, 8.7)	<0.001
LDLC, mmol·L^−1^	1.8 (1.3, 2.7)	2.1 (1.5, 2.7)	0.094
HDLC, mmol·L^−1^	1.16 (0.98, 1.40)	1.19 (1.00, 1.41)	0.725
IBIL, µmol·L^−1^	8.3 (5.2, 11.9)	9.0 (6.4, 12.3)	0.159
ALP, U·L^−1^	80.0 (62, 102.2)	79.5 (63.9, 95.0)	0.853
Globulin, g·L^−1^	27.3 (24.3, 31.0)	27.8 (24.6, 31.2)	0.321
Hematocrit	38.6 (33.5, 41.6)	39.4 (35.7, 42.6)	<0.05
D-dimer, mg·L^−1^	0.70 (0.42, 1.80)	0.43 (0.24, 0.91)	<0.001
TSH, miu·L^−1^	1.98 (1.18, 3.30)	1.82 (1.09, 2.71)	0.314
HS-TNTI, ng·L^−1^	8.2 (3.7, 33.4)	5.2 (2.2, 19.2)	<0.05
Creatinine, µmol·L^−1^	77.9 (63.3, 93.2)	69.9 (59.2, 87.3)	<0.05
Myoglobin, ng·mL^−1^	58.2 (39.0, 92.9)	45.6 (33.7, 69.7)	<0.01
Creatine kinase, U·L^−1^	79 (54, 119)	90 (63, 138)	0.066
Uric acid, µmol·L^−1^	345 (274, 438)	340 (266, 411)	0.402
Total bile acid, µmol·L^−1^	5.4 (3.2, 8.4)	4.2 (2.5, 8.1)	0.303
hsCRP, mg·L^−1^	4.0 (1.0, 28.0)	1.5 (0.5, 7.2)	<0.01
Lymphocyte, *10^9^·L^−1^	1.2 (0.9, 1.6)	1.3 (0.9, 1.7)	0.168
Basophil, *10^9^·L^−1^	0.029 (0.020, 0.040)	0.027 (0.019, 0.039)	0.320
Eosinophil, *10^9^·L^−1^	0.110 (0.052, 0.194)	0.104 (0.059, 0.186)	0.965

Data are presented as the number (%) or median (Q1, Q3). GGT, γ-glutamyl transpeptidase; ALB, albumin; ALT, alanine aminotransferase; TG, triglyceride; eGFR, estimated glomerular filtration rate; AST, aspartate aminotransferase; TC, total cholesterol; TBIL, total bilirubin; TP, total protein; WBC, white blood cell; RBC, red blood cell; HCT, hematocrit; HGB, hemoglobin; LDLC, low-density lipoprotein cholesterol; HDLC, high-density lipoprotein cholesterol; IBIL, indirect bilirubin; ALP, alkaline phosphatase; TSH, thyroid stimulating hormone; HS-TNTI, HIGH-sensitivity cardiac troponin I; hsCRP, hypersensitive C-reactive protein.

### 3.2. Data processing and variable selection

A total of 178 variables were collected, and the variables assignment results are shown in Supplementary Table S1. These variables included three general information (X1–X3), 15 comorbidities (X4–X18), 32 medications (X19–X50), and 128 laboratory tests (X51–X178). Sixty-five variables with missing value >90%, a single value occupying >90%, and a coefficient of variation <0.1 were deleted (Supplementary Table S2). Lasso was used for variable selection of the rest 113 variables, and 83 variables remained in the subsequent study (Supplementary Table S2).

### 3.3. Establishment and evaluation of the model

**Figure 2:**
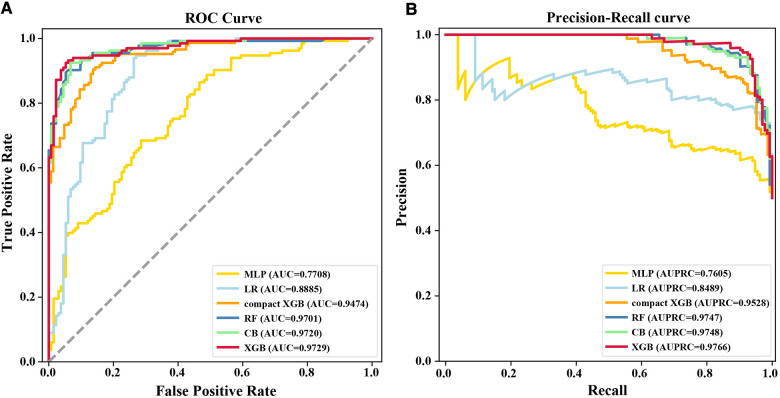
(**A**) ROC curves and (**B**) precision–recall curves for the six ML models on the test set. MLP, multilayer perceptron; AUC, area under the receiver operating characteristic curve; LR, logistic regression; RF, random forest; CB, categorical boosting; XGB, extreme gradient boosting; AUPRC, area under the precision-recall curve.

XGB, MLP, RF, LR, and CB were combined with 83 variables to establish ML models to predict 7-day unplanned readmission in elderly patients with CHD in the training set. The predictive performance of the ML models was checked in the test set. Table 2 presents the AUC, accuracy, precision, recall, and F1 value of the models. Among the models, XGB had the best performance, showing the highest AUC (0.9729), accuracy (0.9173), and F1 value (0.9134). To improve the clinical application of the model, a compact XGB model was applied by the top 10 variables according to the mean absolute SHAP value, which indicates their importance for prediction. The AUC, accuracy, precision, recall, and F1 value of the compact XGB model were 0.9474, 0.8630, 0.9015, 0.8151, and 0.8561, respectively (Table 2). Moreover, the receiver operating characteristic (ROC) curves of the ML models are shown in Figure 2A. In the ML models trained on original data, the CB model had the highest AUC (0.9149). The AUC, accuracy, precision, recall, and F1 value of the XGB model were 0.8446, 0.8443, 0.7500, 0.3529, and 0.4800, respectively (Supplementary Table S3). The ROC curves of the ML models trained on original data are shown in Supplementary Figure S1A.

**Table 2:** Predictive performance of machine learning models on the test set.

Model	AUC	Accuracy	Precision	Recall	F1 value	Brier score
MLP	0.7708	0.5000	0.5000	**1**	0.6667	0.50
LR	0.8885	0.7594	0.8000	0.6917	0.7419	0.24
RF	0.9701	0.8910	**0**.**9727**	0.8045	0.8807	0.11
CB	0.9720	0.9098	0.9360	0.8797	0.9070	0.09
XGB	**0**.**9729**	**0**.**9173**	0.9587	0.8722	**0**.**9134**	**0**.**08**
Compact XGB	0.9474	0.8630	0.9015	0.8151	0.8561	0.14

AUC, area under the receiver operating characteristic curve; MLP, multilayer perceptron; LR, logistic regression; RF, random forest; CB, categorical boosting; XGB, extreme gradient boosting.

Bold values represent the maximum value for each evaluation indicator.

**Figure 3:**
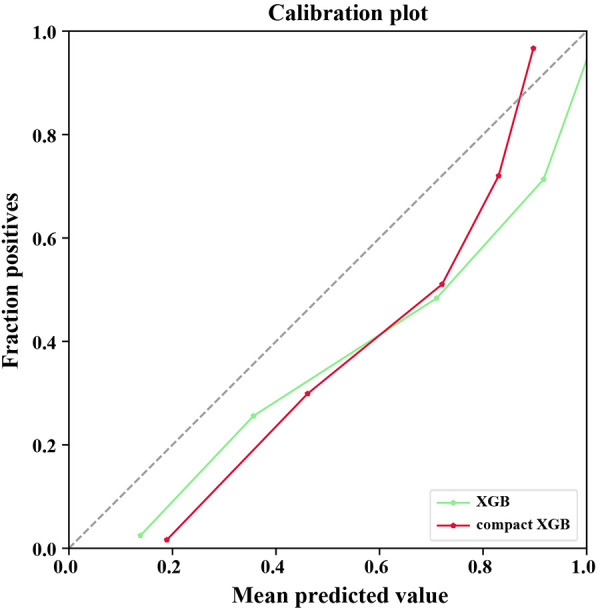
Calibration plot for the XGB and compact XGB models. XGB, extreme gradient boosting.

Recall was defined as the proportion of 7-day unplanned readmission patients who are correctly identified, and precision was defined as the proportion of actual readmission within the predicted readmission patients. For patients and doctors, precision and recall are perhaps clinically more meaningful for the prediction of 7-day unplanned readmission. Therefore, we also used precision-recall curves to evaluate the ML models’ predictive performance (Figure 2B). The result shows that the XGB model had the highest AUPRC (0.9766), and the AUPRC of the compact XGB model was 0.9528. The precision–recall curves of the ML models trained on original data are shown in Supplementary Figure S1B. Meanwhile, we used calibration curves to analyze the calibration ability. The calibration curves of the XGB and compact XGB models showed good calibration performance (Figure 3). The calibration curves of other ML models are shown in Supplementary Figure S2. Furthermore, the Brier score is an index to evaluate both calibration and discrimination of the model. The Brier scores of XGB and compact XGB models were 0.08 and 0.14, respectively (Table 2).

### 3.4. Model interpretation

**Figure 4:**
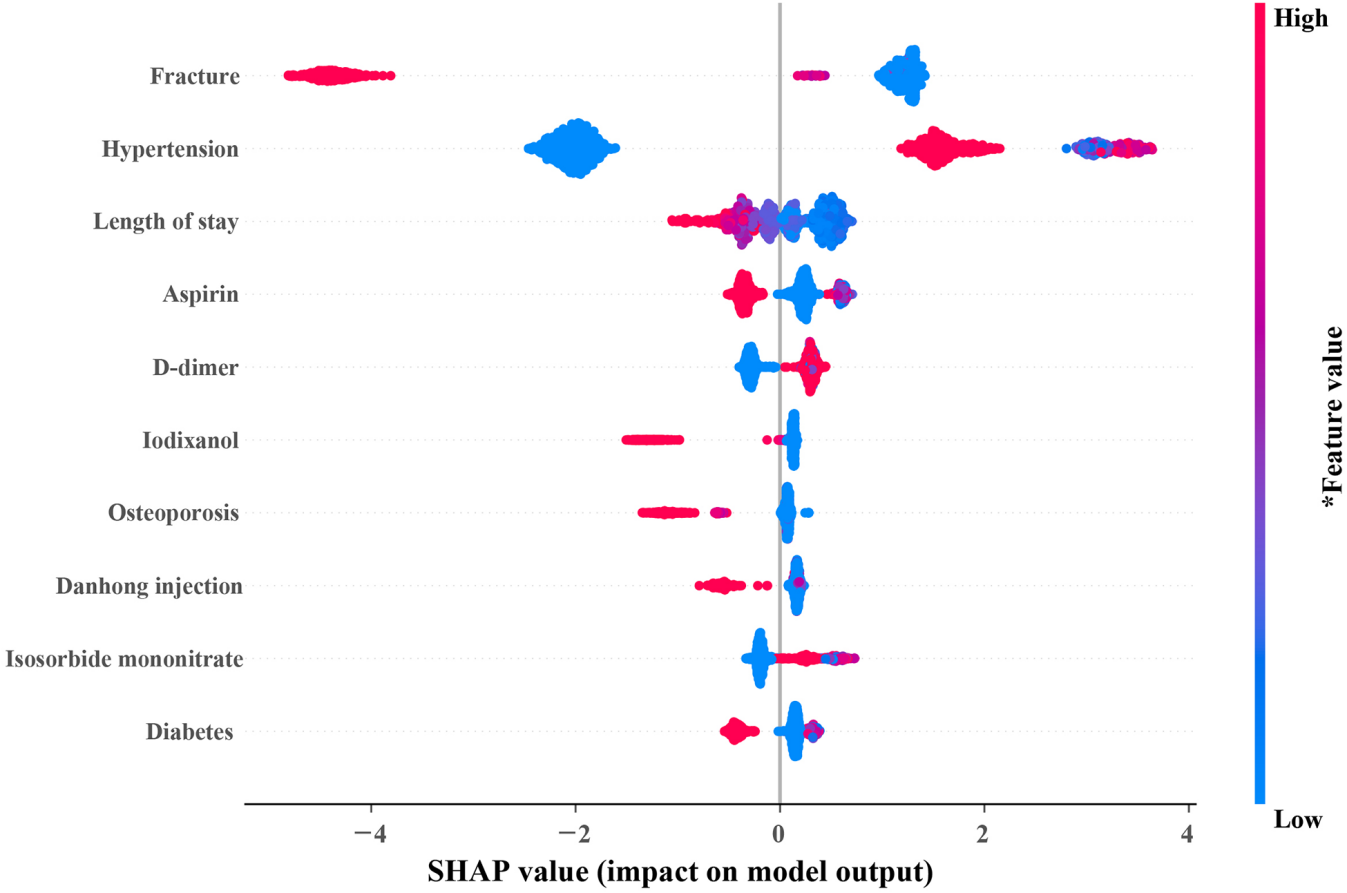
SHAP summary plot of the 10 most important variables of the XGB model. In the SHAP plot, the horizontal axis symbolizes the contribution of variables to the outcome, and the color of the dot represents the value of the variables. Red represents higher variable values, and blue represents lower variable values. SHAP, SHapley additive exPlanations; XGB, extreme gradient boosting.

In Figure 4, the top 10 variables in the XGB model are listed in descending order by the mean absolute SHAP value. The most important 10 variables contributing to the model were fracture, hypertension, length of stay, aspirin, D-dimer, iodixanol, osteoporosis, danhong injection (a traditional Chinese medicine injection used to treat cardiovascular diseases), isosorbide mononitrate, and diabetes. Meanwhile, the 10 variables were used to develop a compact XGB model.

### 3.5. Sample size assessment

**Figure 5:**
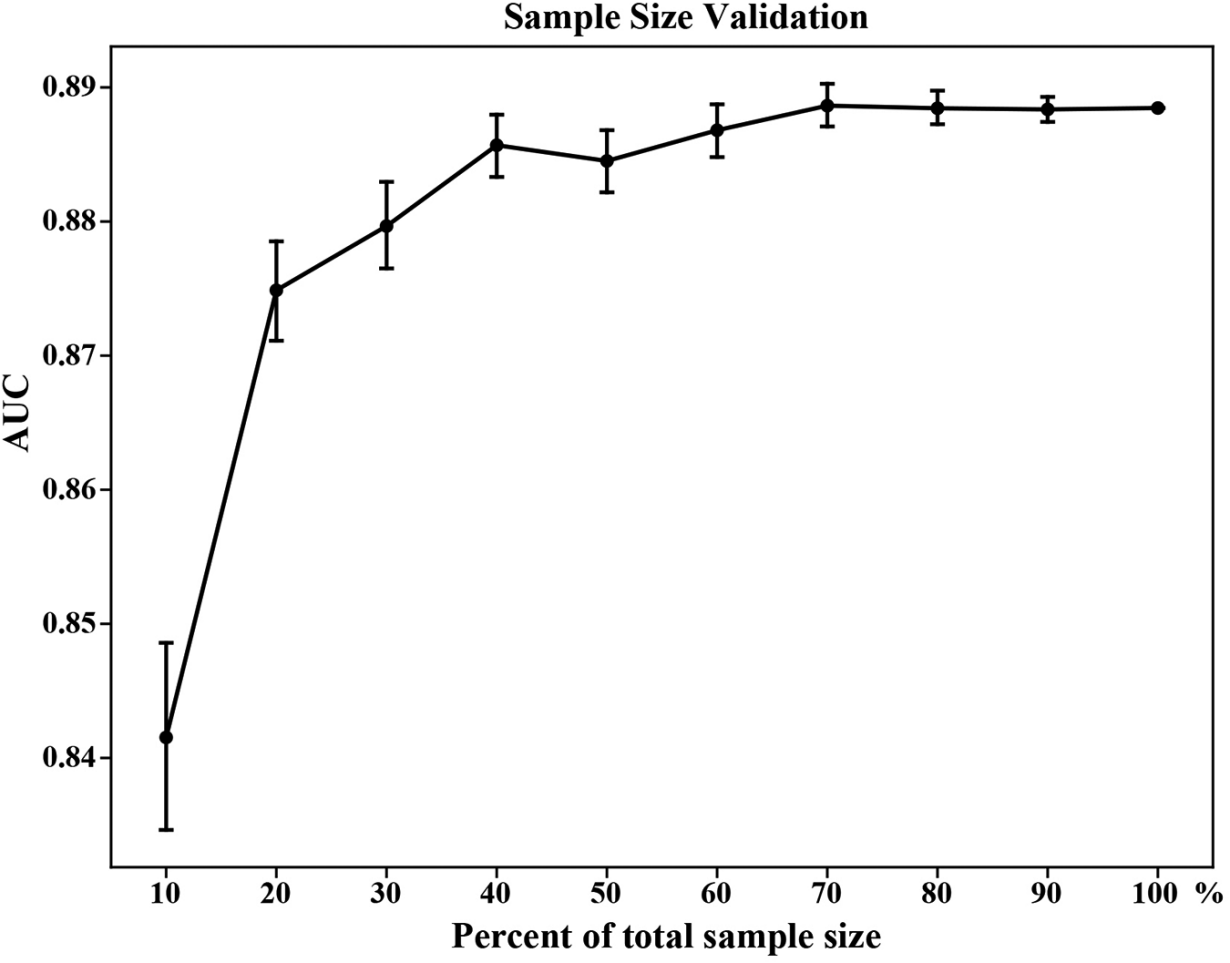
Sample size validation for the XGB model. The vertical bars represent the 95% confidence interval of AUC. XGB, extreme gradient boosting; AUC, area under the receiver operating characteristic curve.

The repeated bootstrapping method was used to assess the adequacy of the sample size, and the result is shown in Figure 5. As the percentage of sample size increased, AUC gradually increased and the fluctuation of the AUC decreased gradually. When the sample size reached 70%, the predictive performance of the model tended to be stable, indicating that a sufficient sample size was included in this study.

## 4. Discussion

In this study, we used XGB, RF, MLP, CB, and LR algorithms, combined with detailed clinical data to establish ML models to predict 7-day unplanned readmission in elderly patients with CHD. We chose XGB as the model with the best fit owing to several factors because it had the highest AUC, accuracy, and F1 value. Although the recall of the MLP model was slightly higher than that of the XGB model, the low accuracy and precision of the MLP model made it difficult to be accepted clinically. In addition, the XGB model had good calibration capability, the best AUPRC, and the best Brier score. Meanwhile, to improve the clinical utility of the model, we used the 10 most important factors to develop a compact XGB model, which also showed a good predictive performance.

Several studies have assessed 30-day or 1-year all-cause readmissions after cardiovascular events (6, 21, 22), but to our knowledge, none focused on 7-day unplanned readmissions in elderly CHD patients. Okere et al. (21) used decision tree algorithms to predict 30-day hospital readmissions of 346,390 hospitalized patients (≥40 years) with a primary diagnosis of ischemic heart disease. The accuracy, precision, recall, and AUC of the model were all above 0.95. However, this study lacked the evaluation of model calibration ability. Gupta and colleagues (6) used 6 ML algorithms, including LR, naïve Bayes, support vector machine, deep neural network, RF, and gradient boosting, to build predictive models of 30-day readmission, but the best C statistic of the model was only 0.641. In China, 30-day readmission is an important indicator to measure the medical quality of third-class hospitals. Some Chinese researchers (22) established nine ML models to predict the risk of 30-day unplanned all-cause hospital readmissions with the AUC in the range of 0.681–0.720. These models could not be used to predict 7-day unplanned readmissions. In fact, 7-day readmission is always neglected, especially for elderly patients with chronic diseases. Therefore, we developed predictive models to accurately identify the elderly CHD patients more likely to be readmitted to the hospital within 7 days.

It is worth noting that the classification model may have a prediction bias. This is because 7-day unplanned readmission is rare and model discrimination is driven by patients without readmission. As a consequence, it is necessary to compare calibration with discrimination simultaneously. In this connection, the Brier score provides a more comprehensive assessment of model performance, combining model discrimination and calibration (23). The Brier score represents the mean square error between the predicted and observed results. Therefore, when two models are compared, the smaller the Brier score, the better the model performance. In our study, the Brier scores of the XGB model were all lower than those of other ML models, which showed that the XGB model had a good calibration ability in predicting 7-day unplanned readmission in elderly patients with CHD.

ML model, called black box frequently, one reason is that it can only offer risk estimates but cannot explain the sources of the risk (24). In recent years, SHAP has been widely used in model interpretation (25, 26). Using the SHAP summary plot, we identified the top contributors to the risk of 7-day unplanned readmission for individual patients. The results of SHAP showed that patients with fractures are unlikely to be readmitted to the hospital in the short term after discharge. Elderly CHD patients with hypertension had higher rehospitalization may due to poor blood pressure control. Moreover, the length of stay has been proven to be an important predictor of readmission of patients with cardiovascular diseases (21, 27, 28), which is consistent with the results of our study. Guidelines routinely recommend aspirin for CHD patients (29). In our study, elderly patients with CHD who did not use aspirin had a higher risk of readmission. This proves that aspirin improves prognosis in elderly patients with CHD. Furthermore, those elderly CHD patients with higher D-dimer had a higher risk of 7-day unplanned readmission because a higher D-dimer meant a higher risk of pulmonary thromboembolism (30, 31).

This study has limitations. First, although we collected the comorbidities of the elderly CHD patients from the EMR, relevant information, such as the severity of the disease and the duration of comorbidities, was not captured in our study and was, therefore, not included in the evaluation. Second, this study was a single-center study, and we were unable to evaluate the performance of the ML models in other medical institutions. Third, due to the retrospective nature of the study, we only collected the basic clinical features. Other features such as socioeconomic factors and health literacy, which may improve the readmission risk assessment, should be validated in further studies. Then, the recall and F1 value declined while the ML models were trained on original data, indicating that a larger sample size is needed to further optimize the model in the future. Finally, a prospective study is needed before the model implements in clinical practice.

## 5. Conclusions

In conclusion, we established ML models to predict 7-day unplanned readmission in elderly patients with CHD using ML algorithms. The XGB model showed the best predictive performance and had good calibration. In addition, the compact XGB model, developed by the top 10 important indicators, can predict 7-day unplanned readmission conveniently. This study showed that an ML-based approach is feasible and effective with a potential clinical application perspective on the reduction of 7-day readmission and the improvement of quality of care to elderly CHD patients.

## Data Availability

The raw data supporting the conclusions of this article will be made available by the authors without undue reservation.

## References

[1] Niu YN Li R Zhao P He P Li YL Wang Y. Quantitative and qualitative research on management strategies for dyspnoea in elderly patients with coronary heart disease complicated with chronic heart failure. J Multidiscip Healthc. (2022) 15:2007–13. 10.2147/jmdh.S378379 36118136 PMC9473661

[2] HealthCare.gov. Hospital readmissions. Available at: https://www.healthcare.gov/glossary/hospital-readmissions/ (Accessed October 10, 2022).

[3] Coatsworth-Puspoky R Duggleby W Dahlke S Hunter K. Unplanned readmission for older persons: a concept analysis. J Adv Nurs. (2021) 77:4291–305. 10.1111/jan.14893 34028852

[4] Rajaguru V Kim TH Han W Shin J Lee SG. LACE index to predict the high risk of 30-day readmission in patients with acute myocardial infarction at a university affiliated hospital. Front Cardiovasc Med. (2022) 9:925965. 10.3389/fcvm.2022.925965 35898272 PMC9309494

[5] Matheny ME Ricket I Goodrich CA Shah RU Stabler ME Perkins AM Development of electronic health record-based prediction models for 30-day readmission risk among patients hospitalized for acute myocardial infarction. JAMA Netw Open. (2021) 4:e2035782. 10.1001/jamanetworkopen.2020.35782 33512518 PMC7846941

[6] Gupta S Ko DT Azizi P Bouadjenek MR Koh M Chong A Evaluation of machine learning algorithms for predicting readmission after acute myocardial infarction using routinely collected clinical data. Can J Cardiol. (2020) 36:878–85. 10.1016/j.cjca.2019.10.023 32204950

[7] Zhao P Yoo I Naqvi SH. Early prediction of unplanned 30-day hospital readmission: model development and retrospective data analysis. JMIR Med Inform. (2021) 9:e16306. 10.2196/16306 33755027 PMC8077543

[8] MedPAC. Chapter 5: Payment policy for inpatient readmissions (June 2007 report). (Accessed October 10, 2022).

[9] Castela Forte J Yeshmagambetova G van der Grinten ML Scheeren TWL Nijsten MWN Mariani MA Comparison of machine learning models including preoperative. Intraoperative, and postoperative data and mortality after cardiac surgery. JAMA Netw Open. (2022) 5:e2237970. 10.1001/jamanetworkopen.2022.37970 36287565 PMC9606847

[10] Sherman E Alejo D Wood-Doughty Z Sussman M Schena S Ong CS Leveraging machine learning to predict 30-day hospital readmission after cardiac surgery. Ann Thorac Surg. (2022) 114:2173–79. 10.1016/j.athoracsur.2021.11.011 34890575

[11] Huang Y Talwar A Lin Y Aparasu RR. Machine learning methods to predict 30-day hospital readmission outcome among US adults with pneumonia: analysis of the national readmission database. BMC Med Inform Decis Mak. (2022) 22:288. 10.1186/s12911-022-01995-3 36352392 PMC9643900

[12] Yuan G Lv B Du X Zhang H Zhao M Liu Y Prediction model for missed abortion of patients treated with IVF-ET based on XGBoost: a retrospective study. PeerJ. (2023) 11:e14762. 10.7717/peerj.14762 36743954 PMC9893909

[13] Chen X Ishwaran H. Random forests for genomic data analysis. Genomics. (2012) 99:323–29. 10.1016/j.ygeno.2012.04.003 22546560 PMC3387489

[14] Abiodun OI Jantan A Omolara AE Dada KV Mohamed NA Arshad H. State-of-the-art in artificial neural network applications: a survey. Heliyon. (2018) 4:e00938. 10.1016/j.heliyon.2018.e00938 30519653 PMC6260436

[15] Mohammadi F Dehbozorgi L Akbari-Hasanjani HR Joz Abbasalian Z Akbari-Hasanjani R Sabbaghi-Nadooshan R Evaluation of effective features in the diagnosis of COVID−19 infection from routine blood tests with multilayer perceptron neural network: a cross-sectional study. Health Sci Rep. (2023) 6:e1048. 10.1002/hsr2.1048 36620509 PMC9817491

[16] Prokhorenkova L Gusev G Vorobev A Dorogush AV Gulin A. CatBoost: unbiased boosting with categorical features. arXiv preprint arXiv:1706.09516 (2017). (2017).

[17] Zabor EC Reddy CA Tendulkar RD Patil S. Logistic regression in clinical studies. Int J Radiat Oncol Biol Phys. (2022) 112:271–77. 10.1016/j.ijrobp.2021.08.007 34416341

[18] Abe D Inaji M Hase T Takahashi S Sakai R Ayabe F A prehospital triage system to detect traumatic intracranial hemorrhage using machine learning algorithms. JAMA Netw Open. (2022) 5:e2216393. 10.1001/jamanetworkopen.2022.16393 35687335 PMC9187955

[19] Chawla NV Bowyer KW Hall LO Kegelmeyer WP. SMOTE: synthetic minority over-sampling technique. J Artif Intell Res. (2002) 16:321–57. 10.1613/jair.953

[20] Lin J Yin M Liu L Gao J Yu C Liu X The development of a prediction model based on random survival forest for the postoperative prognosis of pancreatic cancer: a SEER-based study. Cancers (Basel). (2022) 14:4667. 10.3390/cancers14194667 36230593 PMC9563591

[21] Okere AN Sanogo V Alqhtani H Diaby V. Identification of risk factors of 30-day readmission and 180-day in-hospital mortality, and its corresponding relative importance in patients with ischemic heart disease: a machine learning approach. Expert Rev Pharmacoecon Outcomes Res. (2021) 21:1043–48. 10.1080/14737167.2021.1842200 33131344

[22] Zhang Z Qiu H Li W Chen Y. A stacking-based model for predicting 30-day all-cause hospital readmissions of patients with acute myocardial infarction. BMC Med Inform Decis Mak. (2020) 20:335. 10.1186/s12911-020-01358-w 33317534 PMC7734833

[23] Khera R Haimovich J Hurley NC McNamara R Spertus JA Desai N Use of machine learning models to predict death after acute myocardial infarction. JAMA Cardiol. (2021) 6:633–41. 10.1001/jamacardio.2021.0122 33688915 PMC7948114

[24] The Lancet Respiratory Medicine. Opening the black box of machine learning. Lancet Respir Med. (2018) 6:801. 10.1016/s2213-2600(18)30425-9 30343029

[25] Hyland SL Faltys M Hüser M Lyu X Gumbsch T Esteban C Early prediction of circulatory failure in the intensive care unit using machine learning. Nat Med. (2020) 26:364–73. 10.1038/s41591-020-0789-4 32152583

[26] Lundberg SM Nair B Vavilala MS Horibe M Eisses MJ Adams T Explainable machine-learning predictions for the prevention of hypoxaemia during surgery. Nat Biomed Eng. (2018) 2:749–60. 10.1038/s41551-018-0304-0 31001455 PMC6467492

[27] Miñana G Bosch MJ Núñez E Mollar A Santas E Valero E Length of stay and risk of very early readmission in acute heart failure. Eur J Intern Med. (2017) 42:61–6. 10.1016/j.ejim.2017.04.003 28400077

[28] Reynolds K Butler MG Kimes TM Rosales AG Chan W Nichols GA. Relation of acute heart failure hospital length of stay to subsequent readmission and all-cause mortality. Am J Cardiol. (2015) 116:400–5. 10.1016/j.amjcard.2015.04.052 26037295

[29] Levine GN Bates ER Bittl JA Brindis RG Fihn SD Fleisher LA 2016 ACC/AHA guideline focused update on duration of dual antiplatelet therapy in patients with coronary artery disease: a report of the American College of Cardiology/American Heart Association task force on clinical practice guidelines. J Thorac Cardiovasc Surg. (2016) 152:1243–75. 10.1016/j.jtcvs.2016.07.044 27751237

[30] Bai Z Huang Y Song C Liu H Chen Y Zhang H Clinical application of the innovance D-dimer assay in the diagnosis of acute pulmonary thromboembolism. Exp Ther Med. (2017) 13:3543–48. 10.3892/etm.2017.4400 28587438 PMC5450673

[31] An J Sun B Ji Y Zhang Z Zhai Z Wang C. d-dimer is a predictor of clot resolution in patients with pulmonary thromboembolism: a retrospective cohort study. Clin Respir J. (2020) 14:549–56. 10.1111/crj.13167 32052554

