# Supplementary material for: Predicting 7-day unplanned readmission in elderly patients with coronary heart disease using machine learning
Source: Front Cardiovasc Med. 2023 Aug 8;10:1190038. doi: 10.3389/fcvm.2023.1190038 (PMC10442485; doi:10.3389/fcvm.2023.1190038)

Supplementary Material

Predicting 7-day unplanned readmission in elderly patients with coronary heart disease using machine learning

Xuewu Song^1,2^, Yitong Tong^3^, Yi Luo^1,2^, Huan Chang^1,2^, Guangjie Gao^1,2^, Ziyi Dong^1,2^, Xingwei Wu^1,2^*, and Rongsheng Tong^1,2^*

*** Correspondence:**Xingwei Wu
7190175@uestc.edu.cn

Rongsheng Tong
[318004031@qq.com](mailto:318004031@qq.com)

# Supplementary Tables

TABLE 1 Variable assignment

| Number | Variable | Assignment |
| --- | --- | --- |
| Y | 7-day unplanned readmission | 1, Yes; 0, No |
| X1 | Age (years) | Observed value |
| X2 | Gender | 1, Male; 0, Female |
| X3 | Length of stay (days) | Observed value |
| X4 | Hypertension | 1, Yes; 0, No |
| X5 | Diabetes | 1, Yes; 0, No |
| X6 | Fracture | 1, Yes; 0, No |
| X7 | Stenocardia | 1, Yes; 0, No |
| X8 | Osteoporosis | 1, Yes; 0, No |
| X9 | Hyperlipemia | 1, Yes; 0, No |
| X10 | Cerebral infarction | 1, Yes; 0, No |
| X11 | Heart failure | 1, Yes; 0, No |
| X12 | Hypertensive heart disease | 1, Yes; 0, No |
| X13 | Arrhythmia | 1, Yes; 0, No |
| X14 | Renal insufficiency | 1, Yes; 0, No |
| X15 | Myocardial infarction | 1, Yes; 0, No |
| X16 | Benign prostatic hyperplasia | 1, Yes; 0, No |
| X17 | Nephrocystosis | 1, Yes; 0, No |
| X18 | Anemia | 1, Yes; 0, No |
| X19 | Alprazolam | 1, Yes; 0, No |
| X20 | Aspirin | 1, Yes; 0, No |
| X21 | Atorvastatin | 1, Yes; 0, No |
| X22 | Amlodipine | 1, Yes; 0, No |
| X23 | Ambroxol | 1, Yes; 0, No |
| X24 | Budesonide | 1, Yes; 0, No |
| X25 | Salviae miltiorrhizae and ligustrazine hydrochloride injection | 1, Yes; 0, No |
| X26 | Danhong injection | 1, Yes; 0, No |
| X27 | Isosorbide mononitrate | 1, Yes; 0, No |
| X28 | Dexamethasone | 1, Yes; 0, No |
| X29 | Iodixanol | 1, Yes; 0, No |
| X30 | Irbesartan | 1, Yes; 0, No |
| X31 | Calcii dibutyry-ladenosini cyclophosphas for injection | 1, Yes; 0, No |
| X32 | Furosemide | 1, Yes; 0, No |
| X33 | Potassium citrate | 1, Yes; 0, No |
| X34 | Bismuth agent | 1, Yes; 0, No |
| X35 | Polyethylene glycol | 1, Yes; 0, No |
| X36 | Rabeprazole | 1, Yes; 0, No |
| X37 | Lidocaine | 1, Yes; 0, No |
| X38 | Spirolactone | 1, Yes; 0, No |
| X39 | Clopidogrel | 1, Yes; 0, No |
| X40 | Metoprolol | 1, Yes; 0, No |
| X41 | Trimetazidine | 1, Yes; 0, No |
| X42 | Rosuvastatin | 1, Yes; 0, No |
| X43 | Terbutaline | 1, Yes; 0, No |
| X44 | Ticagrelor | 1, Yes; 0, No |
| X45 | Nifedipine | 1, Yes; 0, No |
| X46 | Nitroglycerin | 1, Yes; 0, No |
| X47 | Isosorbide dinitrate | 1, Yes; 0, No |
| X48 | Valsartan | 1, Yes; 0, No |
| X49 | Enoxaparin | 1, Yes; 0, No |
| X50 | Insulin | 1, Yes; 0, No |
| X51 | 5'-nucleotidase | 1, Below the normal range; 2, Within the normal range; 3, Above the normal range |
| X52 | Creatine kinase muscle and brain isoenzyme (CKMB) | 1, Below the normal range; 2, Within the normal range; 3, Above the normal range |
| X53 | α-l-fucosidase | 1, Below the normal range; 2, Within the normal range; 3, Above the normal range |
| X54 | α-hydroxybutyrate dehydrogenase | 1, Below the normal range; 2, Within the normal range; 3, Above the normal range |
| X55 | γ-glutamyl transpeptidase | 1, Below the normal range; 2, Within the normal range; 3, Above the normal range |
| X56 | Carcinoembryonic antigen | 1, Below the normal range; 2, Within the normal range; 3, Above the normal range |
| X57 | Albumin | 1, Below the normal range; 2, Within the normal range; 3, Above the normal range |
| X58 | Albumin and globulin ratio | 1, Below the normal range; 2, Within the normal range; 3, Above the normal range |
| X59 | Alanine aminotransferase (ALT) | 1, Below the normal range; 2, Within the normal range; 3, Above the normal range |
| X60 | Thyroid stimulating hormone | 1, Below the normal range; 2, Within the normal range; 3, Above the normal range |
| X61 | Cholinesterase | 1, Below the normal range; 2, Within the normal range; 3, Above the normal range |
| X62 | Low density lipoprotein cholesterol | 1, Below the normal range; 2, Within the normal range; 3, Above the normal range |
| X63 | Serum carbon dioxide | 1, Below the normal range; 2, Within the normal range; 3, Above the normal range |
| X64 | Triglyceride | 1, Below the normal range; 2, Within the normal range; 3, Above the normal range |
| X65 | High density lipoprotein cholesterol | 1, Below the normal range; 2, Within the normal range; 3, Above the normal range |
| X66 | High-sensitivity troponin | 1, Below the normal range; 2, Within the normal range; 3, Above the normal range |
| X67 | Estimated glomerular filtration rate | 1, Below the normal range; 2, Within the normal range; 3, Above the normal range |
| X68 | Aspartate aminotransferase (AST)/ALT | 1, Below the normal range; 2, Within the normal range; 3, Above the normal range |
| X69 | Cystatin C | 1, Below the normal range; 2, Within the normal range; 3, Above the normal range |
| X70 | Creatinine | 1, Below the normal range; 2, Within the normal range; 3, Above the normal range |
| X71 | Myohemoglobin | 1, Below the normal range; 2, Within the normal range; 3, Above the normal range |
| X72 | CK | 1, Below the normal range; 2, Within the normal range; 3, Above the normal range |
| X73 | Activity of CKMB | 1, Below the normal range; 2, Within the normal range; 3, Above the normal range |
| X74 | α-fetoprotein | 1, Below the normal range; 2, Within the normal range; 3, Above the normal range |
| X75 | Serum potassium | 1, Below the normal range; 2, Within the normal range; 3, Above the normal range |
| X76 | Indirect bilirubin | 1, Below the normal range; 2, Within the normal range; 3, Above the normal range |
| X77 | Alkaline phosphatase | 1, Below the normal range; 2, Within the normal range; 3, Above the normal range |
| X78 | Procalcitonin test | 1, Below the normal range; 2, Within the normal range; 3, Above the normal range |
| X79 | Antithyroid peroxidase | 1, Below the normal range; 2, Within the normal range; 3, Above the normal range |
| X80 | Antithyroglobulin | 1, Below the normal range; 2, Within the normal range; 3, Above the normal range |
| X81 | Serum phosphatase | 1, Below the normal range; 2, Within the normal range; 3, Above the normal range |
| X82 | Squamous cell carcinoma antigen | 1, Below the normal range; 2, Within the normal range; 3, Above the normal range |
| X83 | Serum magnesium | 1, Below the normal range; 2, Within the normal range; 3, Above the normal range |
| X84 | Urea | 1, Below the normal range; 2, Within the normal range; 3, Above the normal range |
| X85 | Urea/Creatinine | 1, Below the normal range; 2, Within the normal range; 3, Above the normal range |
| X86 | Uric acid | 1, Below the normal range; 2, Within the normal range; 3, Above the normal range |
| X87 | Serum glucose | 1, Below the normal range; 2, Within the normal range; 3, Above the normal range |
| X88 | Prealbumin | 1, Below the normal range; 2, Within the normal range; 3, Above the normal range |
| X89 | Prostate-specific antigen | 1, Below the normal range; 2, Within the normal range; 3, Above the normal range |
| X90 | Globulin | 1, Below the normal range; 2, Within the normal range; 3, Above the normal range |
| X91 | Lactic dehydrogenase | 1, Below the normal range; 2, Within the normal range; 3, Above the normal range |
| X92 | Neuronspecific enolase | 1, Below the normal range; 2, Within the normal range; 3, Above the normal range |
| X93 | Carbohydrate antigen 125 | 1, Below the normal range; 2, Within the normal range; 3, Above the normal range |
| X94 | Carbohydrate antigen 15-3 | 1, Below the normal range; 2, Within the normal range; 3, Above the normal range |
| X95 | Carbohydrate antigen 19-9 | 1, Below the normal range; 2, Within the normal range; 3, Above the normal range |
| X96 | Carbohydrate antigen 242 | 1, Below the normal range; 2, Within the normal range; 3, Above the normal range |
| X97 | Carbohydrate antigen 50 | 1, Below the normal range; 2, Within the normal range; 3, Above the normal range |
| X98 | Carbohydrate antigen 72-4 | 1, Below the normal range; 2, Within the normal range; 3, Above the normal range |
| X99 | AST | 1, Below the normal range; 2, Within the normal range; 3, Above the normal range |
| X100 | Ferritin | 1, Below the normal range; 2, Within the normal range; 3, Above the normal range |
| X101 | Homocysteine | 1, Below the normal range; 2, Within the normal range; 3, Above the normal range |
| X102 | Cytokeratin 19 fragment 21-1 | 1, Below the normal range; 2, Within the normal range; 3, Above the normal range |
| X103 | Adenosine deaminase | 1, Below the normal range; 2, Within the normal range; 3, Above the normal range |
| X104 | Thymidine kinase 1 | 1, Below the normal range; 2, Within the normal range; 3, Above the normal range |
| X105 | Hepatitis B surface antigen (plasma) | 1, Below the normal range; 2, Within the normal range; 3, Above the normal range |
| X106 | Anion gap | 1, Below the normal range; 2, Within the normal range; 3, Above the normal range |
| X107 | Free thyroxine | 1, Below the normal range; 2, Within the normal range; 3, Above the normal range |
| X108 | Free prostate specific antigen | 1, Below the normal range; 2, Within the normal range; 3, Above the normal range |
| X109 | Free triiodothyronine | 1, Below the normal range; 2, Within the normal range; 3, Above the normal range |
| X110 | Apolipoprotein A-1 (ApoA-1) | 1, Below the normal range; 2, Within the normal range; 3, Above the normal range |
| X111 | ApoA-1/ApoB | 1, Below the normal range; 2, Within the normal range; 3, Above the normal range |
| X112 | ApoB | 1, Below the normal range; 2, Within the normal range; 3, Above the normal range |
| X113 | Lipoprotein | 1, Below the normal range; 2, Within the normal range; 3, Above the normal range |
| X114 | Direct bilirubin | 1, Below the normal range; 2, Within the normal range; 3, Above the normal range |
| X115 | Total cholesterol | 1, Below the normal range; 2, Within the normal range; 3, Above the normal range |
| X116 | Total bilirubin (plasma) | 1, Below the normal range; 2, Within the normal range; 3, Above the normal range |
| X117 | Total bile acid | 1, Below the normal range; 2, Within the normal range; 3, Above the normal range |
| X118 | Total protein (plasma) | 1, Below the normal range; 2, Within the normal range; 3, Above the normal range |
| X119 | Total thyroxine | 1, Below the normal range; 2, Within the normal range; 3, Above the normal range |
| X120 | Total triiodothyronine | 1, Below the normal range; 2, Within the normal range; 3, Above the normal range |
| X121 | White blood cell (plasma) | 1, Below the normal range; 2, Within the normal range; 3, Above the normal range |
| X122 | High-sensitivity C-reactive protein | 1, Below the normal range; 2, Within the normal range; 3, Above the normal range |
| X123 | Percentage of monocytes | 1, Below the normal range; 2, Within the normal range; 3, Above the normal range |
| X124 | Monocytes count | 1, Below the normal range; 2, Within the normal range; 3, Above the normal range |
| X125 | Red blood cell distribution width | 1, Below the normal range; 2, Within the normal range; 3, Above the normal range |
| X126 | Red blood cell (plasma) | 1, Below the normal range; 2, Within the normal range; 3, Above the normal range |
| X127 | Hematocrit | 1, Below the normal range; 2, Within the normal range; 3, Above the normal range |
| X128 | Percentage of lymphocytes | 1, Below the normal range; 2, Within the normal range; 3, Above the normal range |
| X129 | Lymphocyte count | 1, Below the normal range; 2, Within the normal range; 3, Above the normal range |
| X130 | C-reaction protein | 1, Below the normal range; 2, Within the normal range; 3, Above the normal range |
| X131 | Percentage of basophils | 1, Below the normal range; 2, Within the normal range; 3, Above the normal range |
| X132 | Basophil count | 1, Below the normal range; 2, Within the normal range; 3, Above the normal range |
| X133 | Percentage of eosinophils | 1, Below the normal range; 2, Within the normal range; 3, Above the normal range |
| X134 | Eosinophils count | 1, Below the normal range; 2, Within the normal range; 3, Above the normal range |
| X135 | Glycosylated hemoglobin | 1, Below the normal range; 2, Within the normal range; 3, Above the normal range |
| X136 | Percentage of reticulocytes | 1, Below the normal range; 2, Within the normal range; 3, Above the normal range |
| X137 | Reticulocytes | 1, Below the normal range; 2, Within the normal range; 3, Above the normal range |
| X138 | Hemoglobin | 1, Below the normal range; 2, Within the normal range; 3, Above the normal range |
| X139 | Platelet count | 1, Below the normal range; 2, Within the normal range; 3, Above the normal range |
| X140 | The percentage of neutrophils | 1, Below the normal range; 2, Within the normal range; 3, Above the normal range |
| X141 | Neutrophil count | 1, Below the normal range; 2, Within the normal range; 3, Above the normal range |
| X142 | B-type natriuretic peptide | 1, Below the normal range; 2, Within the normal range; 3, Above the normal range |
| X143 | D-dimer | 1, Below the normal range; 2, Within the normal range; 3, Above the normal range |
| X144 | The ratio of activated partial thromboplastin time | 1, Below the normal range; 2, Within the normal range; 3, Above the normal range |
| X145 | Activated partial thromboplastin time | 1, Below the normal range; 2, Within the normal range; 3, Above the normal range |
| X146 | Hepatitis B envelop antibody | 1, Below the normal range; 2, Within the normal range; 3, Above the normal range |
| X147 | Hepatitis B surface antibody | 1, Below the normal range; 2, Within the normal range; 3, Above the normal range |
| X148 | Hepatitis B core antibody | 1, Below the normal range; 2, Within the normal range; 3, Above the normal range |
| X149 | Thrombin time | 1, Below the normal range; 2, Within the normal range; 3, Above the normal range |
| X150 | Prothrombin ratio | 1, Below the normal range; 2, Within the normal range; 3, Above the normal range |
| X151 | International normalized ratio of prothrombin time | 1, Below the normal range; 2, Within the normal range; 3, Above the normal range |
| X152 | Prothrombin time | 1, Below the normal range; 2, Within the normal range; 3, Above the normal range |
| X153 | Prothrombin time activity | 1, Below the normal range; 2, Within the normal range; 3, Above the normal range |
| X154 | Plasma glucose | 1, Below the normal range; 2, Within the normal range; 3, Above the normal range |
| X155 | Heat shock protein 90α | 1, Below the normal range; 2, Within the normal range; 3, Above the normal range |
| X156 | Fibrinogen degradation product | 1, Below the normal range; 2, Within the normal range; 3, Above the normal range |
| X157 | Fibrinogen | 1, Below the normal range; 2, Within the normal range; 3, Above the normal range |
| X158 | Hepatitis Be antigen | 1, Below the normal range; 2, Within the normal range; 3, Above the normal range |
| X159 | Hepatitis B surface antigen (serum) | 1, Below the normal range; 2, Within the normal range; 3, Above the normal range |
| X160 | Total bilirubin (serum) | 1, Below the normal range; 2, Within the normal range; 3, Above the normal range |
| X161 | Total protein (serum) | 1, Below the normal range; 2, Within the normal range; 3, Above the normal range |
| X162 | Urine pH value | 1, Below the normal range; 2, Within the normal range; 3, Above the normal range |
| X163 | White blood cell (urine) | 1, Below the normal range; 2, Within the normal range; 3, Above the normal range |
| X164 | White blood cell in high power field (urine) | 1, Below the normal range; 2, Within the normal range; 3, Above the normal range |
| X165 | Urine conductivity | 1, Below the normal range; 2, Within the normal range; 3, Above the normal range |
| X166 | Urine cast | 1, Below the normal range; 2, Within the normal range; 3, Above the normal range |
| X167 | Urine cast in low power field | 1, Below the normal range; 2, Within the normal range; 3, Above the normal range |
| X168 | Red blood cell (urine) | 1, Below the normal range; 2, Within the normal range; 3, Above the normal range |
| X169 | Red blood cell in high power field (urine) | 1, Below the normal range; 2, Within the normal range; 3, Above the normal range |
| X170 | Squamous epithelial cell (urine) | 1, Below the normal range; 2, Within the normal range; 3, Above the normal range |
| X171 | Epithelial cell (urine) | 1, Below the normal range; 2, Within the normal range; 3, Above the normal range |
| X172 | Epithelial cell in high power field (urine) | 1, Below the normal range; 2, Within the normal range; 3, Above the normal range |
| X173 | Yeast-like fungi (urine) | 1, Below the normal range; 2, Within the normal range; 3, Above the normal range |
| X174 | Bacteria (urine) | 1, Below the normal range; 2, Within the normal range; 3, Above the normal range |
| X175 | Bacteria in high power field (urine) | 1, Below the normal range; 2, Within the normal range; 3, Above the normal range |
| X176 | Small red blood cell (urine) | 1, Below the normal range; 2, Within the normal range; 3, Above the normal range |
| X177 | Low-transitional epithelium (urine) | 1, Below the normal range; 2, Within the normal range; 3, Above the normal range |
| X178 | Mucous strands (urine) | 1, Below the normal range; 2, Within the normal range; 3, Above the normal range |

TABLE 2 Results of different variable preprocessing methods

| Method | Included variables |
| --- | --- |
| Column deletion | X1, X2, X3, X4, X5, X6, X7, X8, X9, X10, X11, X12, X13, X14, X15, X16, X17, X18, X19, X20, X21, X22, X23, X24, X25, X26, X27, X28, X29, X30, X31, X32, X33, X34, X35, X36, X37, X38, X39, X40, X41, X42, X43, X44, X45, X46, X47, X48, X49, X50, X55, X57, X58, X59, X61, X62, X63, X64, X65, X66, X67, X68, X69, X70, X72, X75, X78, X81, X83, X84, X86, X87, X91, X92, X99, X100, X101, X102, X106, X110, X111, X112, X113, X115, X116, X118, X121, X122, X124, X125, X126, X127, X128, X129, X135, X138, X140, X141, X142, X143, X145, X146, X147, X148, X151, X152, X153, X156, X157, X163, X168, X171, X177 |
| Lasso | X1, X2, X3, X4, X5, X6, X8, X10, X11, X13, X20, X21, X26, X27, X29, X31, X32, X36, X39, X40, X41, X42, X55, X57, X58, X59, X61, X62, X63, X64, X65, X66, X67, X68, X69, X70, X72, X75, X78, X81, X83, X84, X86, X87, X91, X92, X99, X100, X101, X102, X106, X110, X111, X112, X113, X115, X116, X118, X122, X125, X126, X127, X128, X129, X135, X138, X140, X141, X142, X143, X145, X146, X147, X148, X151, X152, X153, X156, X157, X163, X168, X171, X177 |

Variable names are shown in Supplementary Table 1.

TABLE 3 The predictive performance of machine learning models on the test set (trained on original data).

| Model | AUC | Accuracy | Precision | Recall | F1 value | Brier score |
| --- | --- | --- | --- | --- | --- | --- |
| MLP | 0.8682 | 0.8443 | 0.6818 | 0.4412 | 0.5357 | 0.16 |
| LR | 0.8680 | 0.8443 | 0.6818 | 0.4412 | 0.5357 | 0.16 |
| RF | 0.8847 | 0.8084 | 0.6667 | 0.1176 | 0.2000 | 0.19 |
| CB | 0.9149 | 0.8743 | 0.9333 | 0.4118 | 0.5714 | 0.13 |
| XGB | 0.8846 | 0.8443 | 0.7500 | 0.3529 | 0.4800 | 0.16 |
| compact XGB | 0.8845 | 0.8412 | 0.6400 | 0.4706 | 0.5424 | 0.16 |

AUC, area under the receiver operating characteristic curve; MLP, multilayer perceptron; LR, logistic regression; RF, random forest; CB, categorical boosting; XGB, extreme gradient boosting.

# Supplementary Figures

FIGURE 1 (A) Receiver operating characteristic (ROC) curves and (B) Precision-recall curves for the 6 ML models on the test set (trained on original data). MLP, multilayer perceptron; AUC, area under the receiver operating characteristic curve; LR, logistic regression; RF, random forest; CB, categorical boosting; XGB, extreme gradient boosting; AUPRC, area under the precision recall curve.


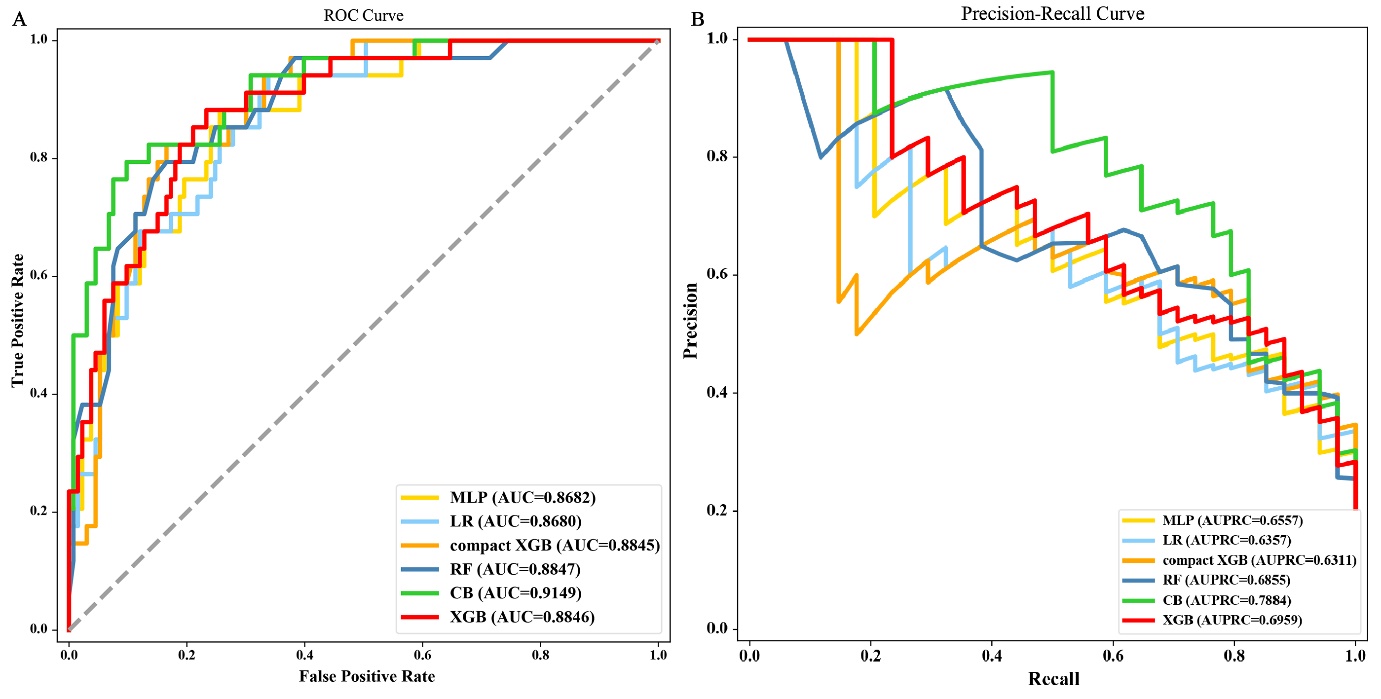


FIGURE 2 Calibration plot of the machine learning models. MLP, multilayer perceptron; LR, logistic regression; RF, random forest; CB, categorical boosting; XGB, extreme gradient boosting.


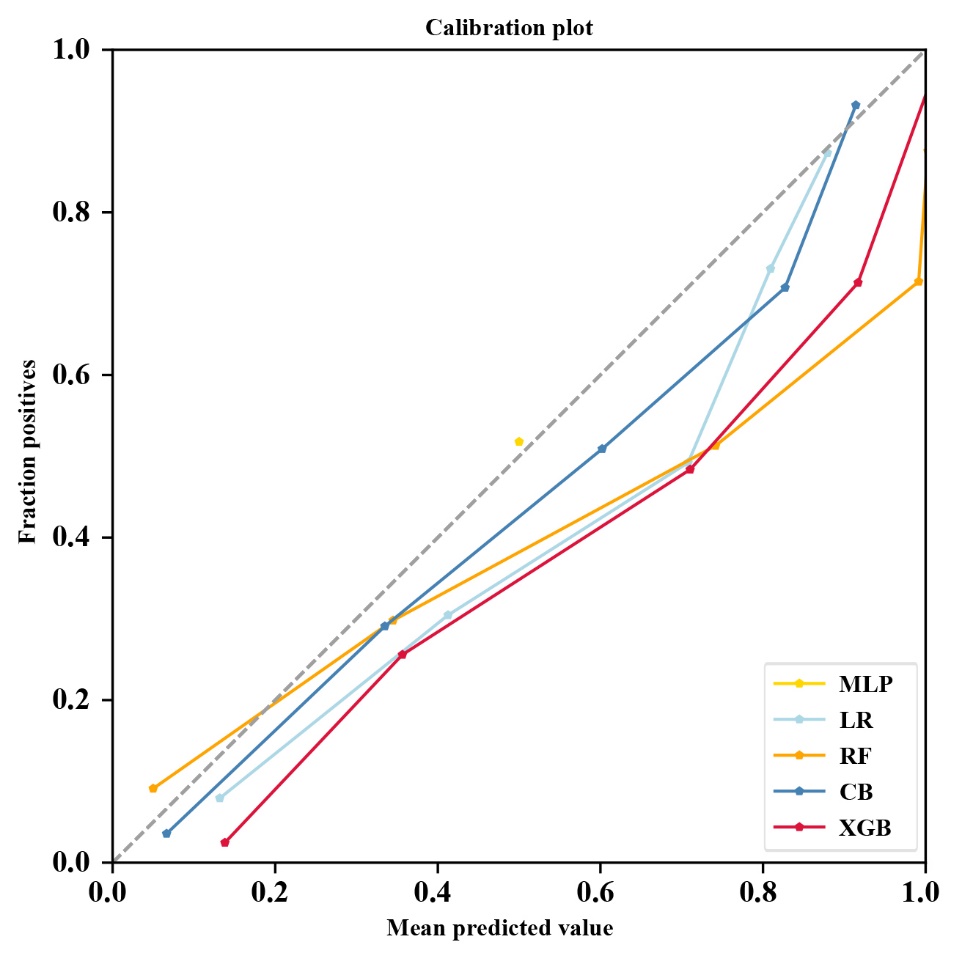

Supplement: Supplementary file 1 [file Table1.docx]
